# Racial Categories in Medical Practice: How Useful Are They?

**DOI:** 10.1371/journal.pmed.0040271

**Published:** 2007-09-25

**Authors:** Lundy Braun, Anne Fausto-Sterling, Duana Fullwiley, Evelynn M Hammonds, Alondra Nelson, William Quivers, Susan M Reverby, Alexandra E Shields

## Abstract

Is it good medical practice for physicians to "eyeball" a patient's race when assessing their medical status or even to ask them to identify their race?

## The Trouble with Race

Is it good medical practice for physicians to “eyeball” a patient's race when assessing their medical status or even to ask them to identify their race? This question was captured in a 2005 episode of “House M.D.,” Fox television's medical drama. In the episode, a black patient with heart disease refuses a hospital physician's prescription for what is clearly supposed to be BiDil, the drug approved by the United States Food and Drug Administration only for “self-identified” African-Americans [1]. Dr. House, on seeing the patient for follow-up, insists on the same prescription. The patient again refuses, telling House, “I'm not buying into no racist drug, OK?” House, a white physician asks, “It's racist because it helps black people more than white people? Well, on behalf of my peeps, let me say, thanks for dying on principle for us.” The patient replies, “Look. My heart's red, your heart's red. And it don't make no sense to give us different drugs.”

Who is right here, House or his patient? And what does this episode tell us about the way race plays itself out in the physician–patient clinical encounter? What of clinical importance can be learned by making a quick racial assessment [2]? That an ACE (angiotensin-converting enzyme) inhibitor may not be effective? That screening for sickle cell anemia is a waste of time? Sorting patients by race may seem useful during a time-constrained interview, but we argue that acting on rapid racial assessment can lead to missed diagnoses and inappropriate treatments.

Both historical evidence and contemporary genetic research suggest that “racial profiling” in medicine can lead to serious medical errors. Assessing risk through race is more problematic than its typical depiction in the media and in scholarly literature [3]. Some argue that race can stand in for human genetic variance until individualized genetic medicine is fully developed. But such a position produces a critical paradox: the rates of morbidity and death from particular diseases are not uniformly distributed among socially defined racial and ethnic groups throughout the world [4–6]. In order to monitor the success of attempts to address these health inequalities, we need to keep health records based on racial and ethnic categories. This is a descriptive use of ethnoracial categories. Descriptive statistics derived from population surveys using racial definitions based on self-identity, however, are not biological or attributive categories appropriate for individual treatment [7]. How should physicians treat individuals who present with a perceived race but who may not bear the average characteristics of a studied population, even while collecting data based on perceived race or ethnicity and qualifying individuals for clinical research trials [8,9]? This problem is illustrated in Box 1, which describes an “on the ground” dilemma of administering a drug to individuals who do not fit “standard” racial categories.

## From Census Categories to Research Plans

Racial categories, with shifting meanings and culturally determined parameters, have always shaped medical practice and thinking, leading to vigorous debates about their use in epidemiology, public health, and medical research journals [10–17]. Throughout the 20th century, race had no standard definition in medical, epidemiological, or health services research [18–21]. In epidemiology, race vaguely referred to “persons who are relatively homogenous with respect to biologic inheritance” [18]. One survey of medical and epidemiological dictionaries found that well into the 1980s definitions assumed that race reflects ”underlying genetic homogeneity” rather than (or even in addition to) shared social experience [22]. Few of the studies making claims for race controlled for socioeconomic status or lifestyle variables.

The embedding of legal and social practices into the “common sense” meaning of race in medical research has been developing for centuries [23]. In the last several decades, poorly defined racial categories became reified in biomedical research practices, in part because of the widespread use of US census categories [24]. Since 2001, NIH-funded researchers have been required to categorize study participants into the five racial or ethnic categories defined by US Office of Management and Budget Directive No. 15 (American Indian or Alaska Native, Asian, Black or African American, Native Hawaiian or other Pacific Islander, or White, and Hispanic/Latino or not Hispanic/Latino) [25]. Thus, state-sanctioned but ill-defined categories of race have entered medical research and practice with the admirable intent of ensuring full racial and gender inclusion in clinical trials, but with unanticipated consequences for health outcomes [26].

Researchers rely on respondents' self-identification to collect data on race and ethnicity. Every research grant must report its study population in these terms, leading to their universal use in recruitment of research subjects. It thus becomes almost “natural” to use these same variables in the subsequent analysis and theoretical framing of the research, even though there is nothing particularly “natural” about the census categories. While the Office of Management and Budget's categories dominate researchers' analyses of human differences in disease, granting agencies' regulations do little to clarify the extent to which racial and ethnic categories are intended to capture biological, cultural, or social dimensions of human diversity. The US Institute of Medicine, for example, holds that race should not be considered a biological reality, but rather “a construct of human variability based on perceived differences in biology, physical appearance, and behavior” [27]. And self-identities are given reality by the very categories we use to describe them [28,29].

Over the past several years, editors at leading medical and scientific journals have promoted a much needed dialogue among researchers and practitioners on the meaning of racial categories [30–40]. The current situation, however, remains confusing. As genetic findings assume an increasingly prominent place in biomedical research, some have concluded that self-identified race/ethnicity, routinely collected in biomedical research studies, is a reasonable proxy for genetic homogeneity and may lead to important insights into health disparities [36,41–43]. Others, citing the genetic heterogeneity within self-identified groups [44–46], argue that race should not be used in genetic research [47–50]. A related perspective comes from those who argue that self-identified racial/ethnic categories may be practical for recruitment into studies, but should not be used in genetic analyses, given that more biologically precise measures of human genetic heterogeneity are available [17,51]. A recent study of geographic patterns of genetic variation, for example, found that “commonly used ethnic labels are both insufficient and inaccurate representations of the inferred genetic clusters, and that drug-metabolizing profiles…;differ significantly among the clusters” [52].

**Box 1. Grace's Dilemma**

“What should I do with my Cape Verdean patient?” insists Grace, a third-year medical student. “The clinical protocol for hypertension requires me to identify his race but I don't know how. Is he black or white? This man immigrated to the US at a young age. Is he now African American or should I consider his health needs from the perspective of his immigrant status?” The data on response to therapy seem to suggest that hypertension in blacks is somehow special, implying a separate genetic factor for blacks. But the enormous national differences in hypertension rates do not support this argument. African Americans suffer at rates 3.5 times those of Nigerians living in Africa, although African Americans experience only 0.75 the rates of Germans in Germany [79]. Which category matters more for Grace's patient, country of origin or social status in the adopted nation?

Physicians everywhere face similar dilemmas. In clinical research projects or in the clinic, the assignment of race assumes an equivalence between census categories and genetics embodied by patients. The large Cape Verdean population in New England resists any simple categorization. The inhabitants are the descendents of Portuguese colonists, former slaves, explorers, and sailors of various nationalities. We suggest that, as with Cape Verdeans, census race cannot be assumed to reflect a particular genetic make-up.

## Racial Categories Are Historical, Not Natural

Historically created racial categories often carry hidden meanings. Until 2003 medical reports were cataloged in PubMed/MEDLINE and in the old Surgeon General's Index Catalogue using 19th century racial categories such as Caucasoid, Monogolid, Negroid and Australoid [53]. Originally suggesting a scale of inferiority and superiority, today such groupings continue to connote notions of human hierarchy [27,54,55]. More importantly, PubMed's newer categories, such as continental population group and ancestry group, merely overlay the older ones. Assuming that “African” origin can capture the complexity of migrations, artificial boundaries, and gene drift is scientifically unsupportable. So too is continued use of the concept of Caucasian (meaning from the central Asian countries surrounding the Caucus Mountains) to emphasize the similarities between disparate European groups rather than their population substructures or variations.

Racial definitions are historically and nationally specific. In her comparison of the history of racial categories in the US and Brazilian census from the late 18th century to the present, political scientist Melissa Nobles demonstrated that categories emerge and are deployed in different ways over time [56]. For example, during the mid-19th to the early 20th centuries, at the height of US anxiety about “miscegenation,” categories such as “mulatto” were vehicles for expressing and containing cultural anxiety about racial purity. Bolstered by scientific ideas about race, data collected on the numbers of “mulattoes” were shaped by the desire to prove that “hybrids” would die out.

Another example of the creation and stabilization of racial categories occurred in the mid-20th century under the apartheid government in South Africa. Obsessed with racial purity, the nationalist government passed the first Population Registration Act in 1950, which defined three groups—coloured, white, and native. According to the Act, a “‘coloured person’ means a person who is not a white person, or a native…‘native’ means a person who in fact is or is generally accepted as a member of any aboriginal race or tribe of Africa…‘white person’ means a person who in appearance obviously is, or who is generally accepted as a white person, but does not include a person who, although in appearance obviously a white person, is generally accepted as a coloured person” [57].

Over the next 30 years, however, numerous amendments attempted to harden those boundaries. By 1967, the definition of a white person was extended to include “his habits, education and speech and deportment and demeanor in general” [58]. Coloured became a residual category, comprised of any person who could not be neatly assigned to one of the two main racial groups. In this definition, as with racial categories in both the United States and Brazil, cultural, class, and biological aspects of human variability are confounded. Since the 1994 dismantling of the apartheid state, racial or “population” categorization remains a subject of discomfort and public debate [59–61]. Categories such as white/European, African, coloured, and Asian, nonetheless, are still widely used in health care settings and in studies of genetic predisposition to disease in South Africa. Differing local conventions in racial categorization present difficulties in transnational collaborative research and peer review in international publications. South African researchers, for example, may feel pressured to employ categories that make no sense in their context (A. Mall, personal communication).

In the early 20th century in the United States, shades of blackness were assumed to affect medical outcomes. This view generated supposed facts (the “fact” that blacks have lesser lung function, for example). Once a “fact” was linked to race rather than unhealthy living and working conditions, it resisted further challenge and became part of clinical judgments. For example, until the widespread use of penicillin, induced malarial fevers were used to treat neurosyphilis and differing malarial strains were deployed based on racial lines [62,63]. As one key 1932 textbook explained, black resistance to tertian malaria could be overcome “the lighter or closer to the Caucasian the particular Negro is” [64,65]. None of the texts explained how to measure color or why it was assumed that blacks, unlike whites, would be exposed to differing malarial strains, as if the mosquitoes respected residential segregation and could not cross a road or tracks. Even though retrospective data made it clear that syphilis was more likely to attack the cardiovascular rather than the neurological system in both blacks and whites, it was assumed that since African Americans did more labor than “brainwork,” they were more at risk for cardiovascular complications. Black cardiovascular deaths, in turn, were often labeled syphilitic in origin without the benefit of autopsy, or misread when postmortems were done (S. M. Reverby, work in progress).

**Box 2. The Future of Research on Race and Health Disparities**

Race-related differences in health outcomes can be analyzed at the societal, individual, cellular, and subcellular levels. Studies focusing exclusively on one level often lose track of inter-level connections. Two research groups exemplify efforts to integrate research on health disparities at the level of cellular effects (e.g., on tumor production and growth), of societal level events (e.g., social support or toxin exposure), and individual life history events (e.g., reproductive history, stress, diet).

Epidemiologist Nancy Krieger [80–82] applies the concept of embodiment to an understanding of how the social effects of racism and social inequality become symptoms [83] and illnesses that manifest as racially related health disparities (on embodiment, see also Fausto-Sterling, 2005 [84]). Krieger understands embodiment to be a multilevel phenomenon that serves as “an antonym to disembodied genes, minds and behaviors” [82]. Similarly, Masi and Olopade [85] propose a multilevel perspective on racial and ethnic disparities in breast cancer. Their model illustrates how the dynamics of societal and individual events over the life cycle can have specific cellular outcomes resulting in neoplasms with particular cellular characteristics. To the extent that societal and individual events vary systematically with social race, biological outcomes may *result from* social inequalities. The key to future understandings of health disparities lies in using frameworks such as those proposed by Krieger and Masi and Olopade to design and interpret research at every level, from the social to the cellular.

## “Knowing” Race: From Research Plans to Individual Treatment

But the debate remains. Even given the history of the (mis)use of racial categories, are they nevertheless useful in the physician's office? Does a quick administrative assessment of race help to diagnose a presenting ailment, or accurately assess future risk of illness? Environmental exposures, family histories, the stress of dealing with racism, access to and quality of care may be left unexamined if a physician simply diagnoses “race” [66]. In the United States a rule that assumes “one drop” of African blood defines an individual as African American [28] seems to prevail [67]. Presented with a black patient, in the face of medical uncertainty, rather than applying individual analysis the doctor can fall back upon general statements that derive from population studies, such as “You should get tested for glaucoma because you are African American and African Americans have a higher rate of glaucoma.”

A dark-skinned, curly-headed person who identifies as African American may, indeed, have much in his or her history and upbringing to justify that identification. But he or she may also have a white grandparent and several Cherokee ancestors. Thus, returning to the example of glaucoma, it is more important to know a patient's family history than to assess his or her race. And collecting family history ought to mean not only compiling a list of which diseases family members have, but making some attempt to assess common (familial) habits such as diet and life experiences (e.g., first- versus second-generation immigrants, living conditions, or same versus widely varied work experience and geographical locations). Similarly, when the history of passing for white is ignored, those who identify themselves as “white” are assumed to have no ancestral “black blood.” Finally, immigration patterns constantly change. A “black” person walking into a Boston, Massachusetts clinic could easily be the child of a recent immigrant from Ethiopia or Brazil who has a genetic makeup as well as cultural and environmental exposures that differ significantly from the descendents of 19th century US slaves from the western coast of Africa [68,69].

Once race is presumed, the ways in which multiple genetic inheritances interact with the environment within that individual seem to disappear (see Box 2). Clinical clues can become invisible. Even with the relatively few diseases “known” to have a 1:1 relationship between a single mutant allele and a disease phenotype, reliance on a general idea of race can lead to misdiagnosis. In a different American television series, ER, a “white” patient with sickle cell anemia was misdiagnosed because the condition is known as a “black” illness. Sickle cell anemia (homozygous HbSS) results from a genetic alteration affecting the hemoglobin protein. Its high prevalence in some populations bespeaks their historical burden of falciparum malaria. The simple gene change responsible for sickle hemoglobin spans the continent of Africa and beyond. Its prevalence in the sub-Saharan region ranges between 10% to 40% [70]. Within even smaller geographical areas this diversity is also apparent. In the tiny West African country of The Gambia, the Mandinka people have an extremely low incidence at 4%, the Wolof are nearly on par with black Americans at 14%, and the more socially endogenous Fula hover just below 30% [71]. Nonetheless, in a clinical encounter in North America, where census category definitions of race prevail, these groups and their descendents would, most likely, occupy the category “Black or African American.” Moreover, some of the highest rates in the world are found in India, with rates of 33% and 35% in the Pardhan and Oktar people, respectively [72]. Sickle cell disease is thus not “race-bound.”

So what is the practicing physician to do? In the case of sickle cell disease, it would be best to work from symptoms rather than racial assumptions, and to enquire about geographic ancestry since sickle cell is more prevalent in populations from the Mediterranean region, sub-Saharan Africa, and the Indian subcontinent [70].

## Is Cultural Competency the Answer?

Clinicians will make better educated patient evaluations if they familiarize themselves with the history of the particular communities they serve. For the clinical encounter, the cultural competency paradigm is sometimes offered as a tool for improving quality of care. Cultural competency advocates have spurred curricular reform so that clinicians in training learn to be attentive to cross-cultural issues. A cultural competency paradigm has recently been suggested as a powerful tool in the arsenal to combat the prevalence of racial and ethnic health disparities [73]. However, when not thoughtfully executed, the cultural competency paradigm can abet the simplistic thinking on race it seeks to address. On the one hand, this perspective brings greater attention to the attitudes and behaviors that patients may bring to the clinical encounter. On the other hand such cultural stereotyping could produce poor health outcomes if the clinician is more attentive to what he or she *thinks* they know about this “type” of patient than to the individual before them [74].

## Race in the Era of Individually Tailored Treatment

Medical researchers want tools that will allow physicians to understand how the individual biosocial system represented by a patient standing before them has either produced symptoms, or has a certain future likelihood of doing so. Whether or not the recent announcement of a $10 million cash award for the first team to sequence 100 genomes in 10 days will get us closer to individual genomic medicine remains to be seen [75]. But in the meantime, race remains a social characteristic of populations and it is inappropriate to use it as a central diagnostic tool for an individual patient.

The case of BiDil, the drug the fictional Dr. House prescribed to his skeptical African American patient, stands as a cautionary tale [76]. Depending on how the age-specific morbidity data on heart disease are read, the case for the urgency of additional treatments for African Americans can be made. Those advocating for BiDil argued that the dangers of the disease are so grave that there was a moral necessity for a race-specific drug, while others found the statistical case for differential morbidity to be unconvincing [77]. By primarily relying on a clinical trial that only included black men and women, claims were made that the drug worked for those who defined themselves as African American. Further, earlier studies that purported to show that ACE inhibitors—another medication for heart failure and an alternative to the active therapies in BiDil—did not work as well on blacks failed to acknowledge that this was not true for all black people in the study [51]. Other researchers who work on drug metabolizing enzymes have argued bluntly that “skin pigment is a lousy surrogate for drug-metabolism status or most any aspect of human physiology ” [51].

BiDil's real impact may therefore be less on actual patient care (since physicians are being encouraged to use the drug “off-label” for anyone they please) and more on the fact that the US government gave its stamp of approval for what bioethicist Sandra Soo-Jin Lee labels “racial profiling in biomedicine” [78]. Although the drug may reify race, this may not be a useful guide to determine who needs it. In the end Dr. House may be right about how medicine is practiced and how drugs are marketed, but his patient understands more about the underlying biology.

## Thoughts for the 10-Minute Clinical Encounter

Improved medical training about race can sharpen diagnostic skills. Cultural competency instruction should be modified to include information on the history of racial categories, current controversies about their biological significance, and the limits of their utility. A teaching unit on race would also contrast the differences between race as a population concept with its meaning when applied to the lives of individuals. In this context it would be appropriate to teach about geographical variations in specific allele frequencies for genes linked to particular disease processes, as well as the cultural practices, historical trends, and environmental conditions that favor their prevalence or not.

Physicians face huge demands for time efficiency and product output, often being called upon to process as many as six patients per hour. No wonder that rapid racial assessment is an attractive means to figure out what to do with a presenting patient. But we argue that even if there are short cuts for the medical interview, race is not a good one. There is, in the end (in addition to noting physical symptoms), no substitute for an inquiry into family history, an assessment of current circumstances, and knowledge about the biological and cultural histories of specific populations serviced by a particular treatment center.

## What Is To Be Done?

In the long run, the problem of whether or how to use race as a diagnostic aid and research category requires an international consensus meeting with representatives from all the biomedical fields. Such a meeting should be organized by the US National Institutes of Health, the World Health Organization, and other international health institutes. In the short run, the National Institutes of Health needs to re-examine its race-based research rules, weighing the balance between attempting to include minority populations in our health care system, on the one hand, without forcing us into a misconstrual of race as biology on the other. Medical courses also need to improve the teaching of the complexities of using race in the clinic. The overall goal of such an effort would be to make clear that “For meaningful statements to be made about health disparities, careful consideration must be given to the way in which race and ethnicity are conceptualized, the choice of definition categories, and the way in which individuals are assigned to categories” [66]. Anthropologist Michael Montoya's distinction between using ethnoracial categories in a *descriptive* mode, to document progress in the health status of populations, but not using basically social categories to produce biological *attribution* of causes will be an essential part of this effort [7]. In the end we have to be able to answer the patient's question—if all hearts are red then why do we need different drugs for different individuals based on race? To provide the best health care we must be able to say why and when race matters and why and when it doesn't.
